# TRAP1 downregulation in human ovarian cancer enhances invasion and epithelial–mesenchymal transition

**DOI:** 10.1038/cddis.2016.400

**Published:** 2016-12-15

**Authors:** Maria R Amoroso, Danilo S Matassa, Ilenia Agliarulo, Rosario Avolio, Haonan Lu, Lorenza Sisinni, Giacomo Lettini, Hani Gabra, Matteo Landriscina, Franca Esposito

**Affiliations:** 1 Dipartimento di Medicina Molecolare e Biotecnologie Mediche, Università di Napoli 'Federico II', Napoli, Italy; 2 Imperial College London, Ovarian Cancer Action Research Centre, Department of Cancer and Surgery, Institute of Reproductive and Developmental Biology, London, UK; 3 Laboratorio di Ricerca Preclinica e Traslazionale, IRCCS-CROB, Centro di Riferimento Oncologico Della Basilicata, Rionero in Vulture, Italy; 4 Ovarian Cancer Action Research Centre, Department of Surgery and Cancer, Imperial College London, London, UK; 5 Dipartimento di Scienze Mediche e Chirurgiche, Università Degli Studi di Foggia, Foggia, Italy

## Abstract

Ovarian cancer (OC) is the second leading cause of gynecological cancer death worldwide. Although the list of biomarkers is still growing, molecular mechanisms involved in OC development and progression remain elusive. We recently demonstrated that lower expression of the molecular chaperone TRAP1 in OC patients correlates with higher tumor grade and stage, and platinum resistance. Herein we show that TRAP1 is often deleted in high-grade serous OC patients (*N*=579), and that TRAP1 expression is correlated with the copy number, suggesting this could be one of the driving mechanisms for the loss of TRAP1 expression in OC. At molecular level, downregulation of TRAP1 associates with higher expression of p70S6K, a kinase frequently active in OC with emerging roles in cell migration and tumor metastasis. Indeed, TRAP1 silencing in different OC cells induces upregulation of p70S6K expression and activity, enhancement of cell motility and epithelial–mesenchymal transition (EMT). Consistently, in a large cohort of OC patients, TRAP1 expression is reduced in tumor metastases and directly correlates with the epithelial marker E-Cadherin, whereas it inversely correlates with the transcription factor Slug and the matrix metallopeptidases 2 and 9. Strikingly, pharmacological inhibition of p70S6K reverts the high motility phenotype of TRAP1 knock-down cells. However, although p70S6K inhibition or silencing reduces the expression of the transcription factors Snail and Slug, thus inducing upregulation of E-Cadherin expression, it is unable to revert EMT induced by TRAP1 silencing; furthermore, p70S6K did not show any significant correlation with EMT genes in patients, nor with overall survival or tumor stage, suggesting an independent and predominant role for TRAP1 in OC progression. Altogether, these results may provide novel approaches in OC with reduced TRAP1 expression, which could be resistant to therapeutic strategies based on the inhibition of the p70S6K pathway, with potential future intervention in OC invasion and metastasis.

Epithelial ovarian cancer (OC) is the second leading cause of gynecological cancer death in developed countries, accounting for 4% of deaths of cancer in women.^1^ OC is considered a chemoresponsive neoplasm, with initial response rates to systemic chemotherapy, exceeding 80% when integrated with primary cytoreductive surgery.^2^ Despite this, the majority of patients achieving a complete response with front-line chemotherapy ultimately develop recurrent disease, with over 50% of women diagnosed with OC eventually dying from their disease within 5 years from diagnosis.^3^ Data from major trials report that the median progression-free survival for patients with advanced disease ranges between 16 and 23 months, while the median overall survival lies between 31 and 65 months.^4^ In this context, progresses in understanding the intricate molecular mechanisms driving OC progression are crucial. Among these, the PI3K/Akt and mTOR/p70S6K pathway is frequently deregulated in OC.^5^ More specifically, PIK3CA, the gene encoding the p110a catalytic subunit of PI3K, is increased in copy number in 40% of OC;^6^ AKT1 and AKT2 are both activated in a large number of OC, with the activation being associated with high-grade tumors and aggressive clinical behavior;^7^ constitutive activation of p70S6K occurs significantly more often in malignant OC than in benign or borderline lesions.^8^ In addition, recent findings show that p70S6K may be involved in several aspects of OC progression, including invasion and metastasis ^9, 10^ and epithelial–mesenchymal transition (EMT), through the induction of Snail,^11^ a transcriptional factor that, in turn, along with other members of the same family such as Slug,^12^ regulates the expression of molecules involved in cell adhesion and invasion, such as E-Cadherin and the matrix metallopeptidase 2 (MMP2) and 9 (MMP9).^13^ Finally, it has been shown that p70S6K is a critical regulator of the actin cytoskeleton in the acquisition of the metastatic phenotype and its depletion or the inhibition of its activity reduced migration.^14^ These observations suggest a significant role for p70S6K in OC. Notably, AKT and p70S6K undergo translational regulation by TRAP1 in colorectal cancers ^15, 16^ and such regulation affects cell migration in HEK293 cells,^16^ in which TRAP1 knock-down yields a higher migratory potential. Although current literature mainly agrees that TRAP1 activity has important implications for neoplastic progression,^17^ data from the different groups only partially overlap, suggesting that TRAP1 may have complex and possibly contextual effects on tumorigenesis.^18^ In fact, TRAP1 has an important role in the progression from a localized to a metastatic disease^19^ and lower TRAP1 levels correlated with increased overall survival^20^ in colorectal cancers, whereas high TRAP1 expression correlates significantly with favorable chemotherapy response and longer overall survival in OC.^21^ Consistently, we have recently shown that TRAP1 expression inversely correlates with grade and stage and directly correlates with survival in a large cohort of OC patients, and that low TRAP1 in OC cells and tissues associated with a metabolic shift, ultimately causing the onset of resistance to cisplatin-based chemotherapy.^22^ Herein, we show that TRAP1 is often deleted in high-grade serous OC and that TRAP1 expression is correlated with the copy number. This can be one of the mechanisms for the downregulation of TRAP1 in higher stage patients, with consequent selection of drug resistant cells. Remarkably, TRAP1 expression is inversely correlated with p70S6K expression and phosphorylation and with genes involved in EMT, thus affecting migration of OC cells and contributing to the acquisition of metastatic phenotype in OC.

## Results

### Loss of TRAP1 expression in OC is due to genetic deletion

**Figure 1:**
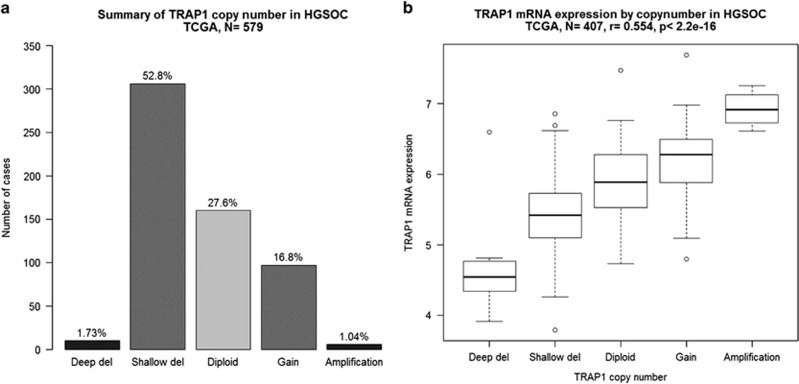
TRAP1 loss of expression in OC. According to data from the TCGA data set, TRAP1 is often deleted in HGSOC (**a**) and TRAP1 expression is correlated with the copy number (**b**) both conditions could account for the downregulation of TRAP1 in higher stage patients, suggesting a possible driver mechanism for TRAP1 loss of expression in OC

Recent findings demonstrate that TRAP1 expression has a positive impact on response to therapy and survival of patients in OC.^21, 22^ To uncover possible mechanisms involved in the decreased TRAP1 levels in higher grade tumors, we analyzed The Cancer Genome Atlas (TCGA) database and found that there is a high percentage of TRAP1 deletion events in high-grade serous OC (Figure 1a) and that TRAP1 expression is correlated with the copy number (Figure 1b). The association between TRAP1 deletion and loss of TRAP1 gene expression suggests that these deletion events, even if 'shallow deletion' (see Materials and Methods for further definition), are enough to have phenotypic effect. Of note, owing to the threshold used, most gene-level copy number variations (CNVs) in high-grade serous OC are actually defined as 'shallow deletion'. Indeed, extensive CNVs are not infrequent in these tumors; however, the association between copy number of TRAP1 and its gene expression suggests this could be one of the driving mechanisms of TRAP1 decrease in OC. These data further support and back up our previous findings showing that TRAP1 expression inversely correlates with grade and stage in OC,^22^ and provide a step forward on the role of TRAP1 in OC progression.

### TRAP1 level is correlated with lower p70S6K

**Figure 2:**
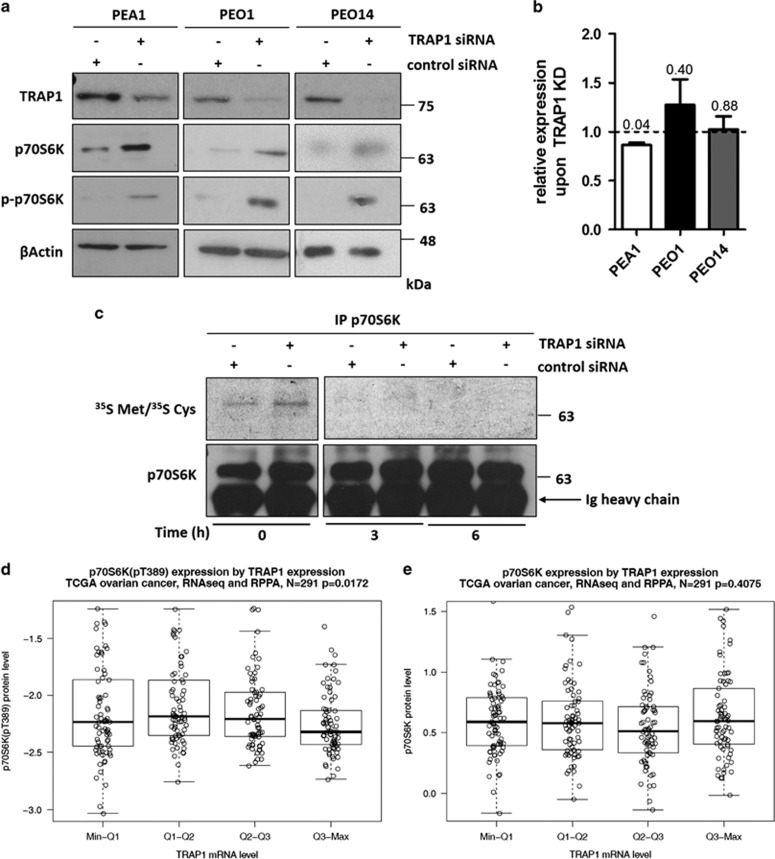
TRAP1 level is correlated with lower p70S6K. (**a**) Total lysates obtained from PEA1, PEO1 and PEO14 cells were separated by SDS-PAGE and immunoblotted with the indicated antibodies. Images are representative of three independent experiments. (**b**) Real-time RT-PCR analysis of p70S6K mRNAs expression in PEA1, PEO1 and PEO14 cells upon siRNA-mediated TRAP1 silencing. Data are expressed as mean±S.E.M. from three independent experiments with technical triplicate each. Numbers above bars indicate the statistical significance (*P*-value), based on the two-tailed Student's *t*-test. Dashed line indicate expression level of the relative control siRNA-transfected cells. (**c**) PEA1 cells were transfected for 60 h with nontargeting control siRNA or TRAP1-directed siRNA, pulse labeled for 1 h as described in Materials and methods and chased for the indicated times. Immunoprecipitates (IP) were analyzed by autoradiography and WB. (**d** and **e**) Correlation between TRAP1 mRNA expression and phospho-p70S6K(Thr389) (**d**) and p70S6K protein expression (**e**). Statistical analysis by Spearman's rank correlation coefficient

To analyze functional consequences of altered TRAP1 expression in cancer models, we further characterized our previous observations showing reduced protein expression and phosphorylation of p70S6K in high TRAP1 background in human colorectal cancer cells and tissues.^15^ PI3K/AKT/p70S6K pathway is frequently activated in human OC, in which TRAP1 has a role in response to therapy and disease progression.^22^ Therefore, starting from these findings, we investigated the role of TRAP1 in the regulation of the p70S6K in OC. Results show that siRNA-mediated silencing of TRAP1 leads to increased expression and phosphorylation levels of p70S6K in three different human OC cell lines (PEA1, PEO1 and PEO14) (Figure 2a). To exclude off-target effect, a second TRAP1 siRNA sequence was used in PEA1 cells, with consistent results (Supplementary Figure 1). qPCR analyses demonstrate that this regulation occurs at a post-transcriptional level, being the mRNA levels not significantly changed upon TRAP1 silencing in the same cell lines (Figure 2b). To further investigate on this regulation, pulse-chase experiments have been performed and show that TRAP1 expression does not affect protein stability as well, whereas a translational regulation is observed (Figure 2c): in fact, p70S6K is already increased in TRAP1-silenced PEA1 cells after 1 h of pulse labeling with radioactive Met/Cys, and such difference is conserved through 3-h chase, as confirmed by detectable signals only in TRAP1-silenced cells. This is consistent with our previous results, showing that TRAP1 exerts a co-translational protein quality control on its substrates.^23^ Remarkably, TRAP1 mRNA level significantly correlated with lower phospho-p70S6K (pT389) in a large data set of OC specimens^24^ (Figure 2d). Interestingly, although weak, an inverse correlation between TRAP1 expression and p70S6K protein levels is also present in patients (Figure 2e). To exclude the possibility that p70S6K could contribute to such inverse correlation by reciprocally regulating TRAP1, we verified TRAP1 expression following p70S6K silencing or treatment with the specific inhibitor PF4708671.^25^ Data show that neither TRAP1 protein (Supplementary Figures 2A and B) nor mRNA expression (Supplementary Figure 2C) are influenced by p70S6K inhibition or siRNA-mediated silencing.

### TRAP1 inversely correlates with OC cell motility

**Figure 3:**
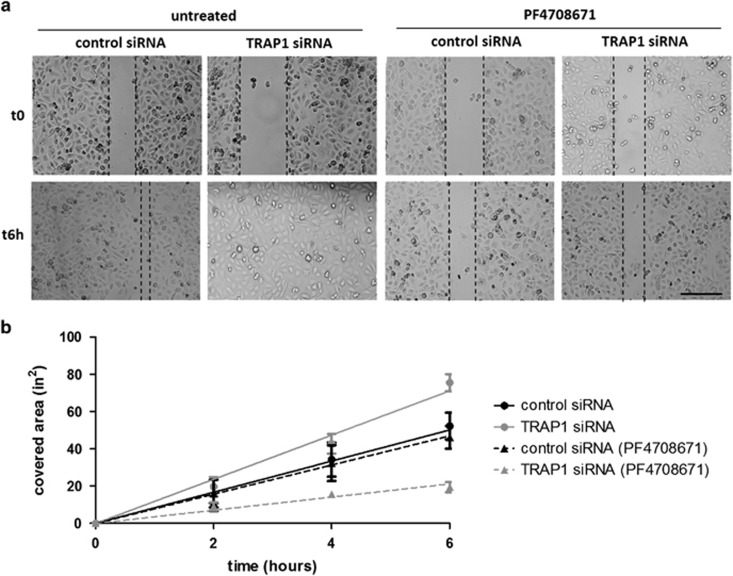
TRAP1 affects cell migration through p70S6K. (**a** and **b**) Time-lapse acquisition of untreated and PF4708671-treated PEA1 cells transfected for 72 h with non-targeted control siRNA or TRAP1-directed siRNA, immediately after the wound (t0) and after 6 h (t6h). Scale bar, 500 *μ*m. The rate of advancement, evaluated as the difference between empty space at each time point, is reported as a function of time in (**b**) for cells transfected with control siRNA (black) and TRAP1-directed siRNA (gray). Dashed lines are used for PF4708671-treated cultures, solid lines for untreated cultures. Data are expressed as mean±S.E.M. from three independent experiments. Lines have been fitted with linear regression

Considering the involvement of TRAP1 in cell migration,^16, 26^ and the functional role played by p70S6K, especially in OC, through the control of actin cytoskeleton dynamics,^14^ we analyzed the migratory behavior of OC cells upon TRAP1 silencing. Wound-healing assays performed in PEA1 OC cells demonstrated that TRAP1 silencing significantly enhances (linear regression F=4.33; *P*=0.05) cell motility (Figures 3a and b): in fact, PEA1 cells transfected with TRAP1-directed siRNAs (Figure 3b, solid gray line) migrate faster than their control siRNA-transfected counterpart (Figure 3b, solid black line). To dissect the role of the p70S6K upregulation consequent to TRAP1 silencing, we treated either control or TRAP1-silenced cells, with the specific p70S6K inhibitor PF4708671.^25^ Results show that the treatment significantly reduces (linear regression F=72.83; *P*<0.01) the rate of edge advancement of TRAP1-silenced PEA1 cells (Figure 3b, dashed gray line), whereas does not significantly (linear regression F=0.53; *P*=0.47) reduce the rate of edge advancement of control cells (Figure 3b, dashed black line), thus leveling off the two migration rates. To exclude off-target effects, the same experiments was performed with a different TRAP1-directed siRNA sequence, yielding similar results (Supplementary Figure 3). As demonstrated in other experimental systems,^16^ treatment with LY294002, a PI3K inhibitor, markedly reduces wound-healing progression in both TRAP1-silenced cells (Supplementary Figure 3B, dotted gray line) and control cells (Supplementary Figure 3B, dotted black line), thus confirming the critical role played by PI3K in the regulation of cell motility in both control and TRAP1-interfered cells.^16^ The significant reduction of phospho-(active) p70S6K confirms the efficacy of both treatments (Supplementary Figure 3C). In parallel experiments, transwell migration assays confirmed that TRAP1 silencing in OC cells enhances cell motility in a p70S6K-dependent manner (Supplementary Figure 4). These results are consistent with those found in other cell lines, linking TRAP1 role in cell metabolism and migratory capacity.^27^

### TRAP1 is inversely correlated with EMT genes

**Figure 4:**
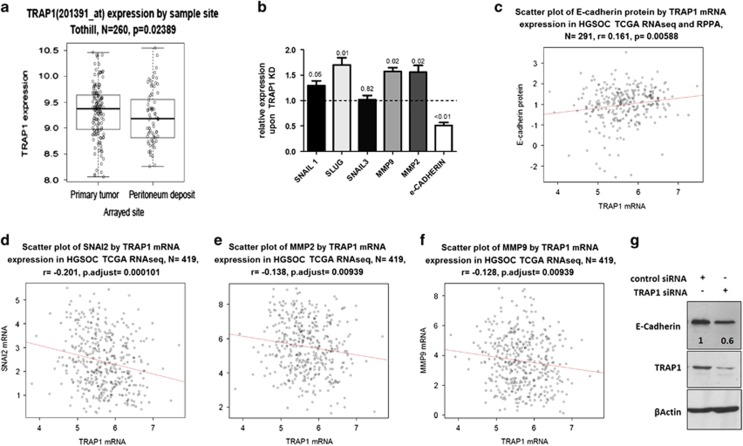
Lower TRAP1 expression is associated with more advanced disease and EMT. (**a**) Correlation between TRAP1 mRNA expression and tumor sample site from the Tothill data set (*P*-values determined by the Wilcoxon rank sum test). (**b**) Real-time RT-PCR analysis of *SNAIL, SNAI2/SLUG, SNAIL3, MMP9, MMP2* and *E-Cadherin* expression in PEA1 cells 72 h after transfection with non-targeted control siRNA or TRAP1-directed siRNA. Data are expressed as mean±S.E.M. from three independent experiments with technical triplicate each. Numbers above bars indicate the statistical significance (*P*-value), based on one-sample *t*-test. Dashed line indicate expression level of the relative control siRNA-transfected cells. (**c**) Correlation between TRAP1 mRNA expression and E-CADHERIN protein expression. Statistical analysis by Spearman's rank correlation coefficient. (**d**–**f**) Correlation between TRAP1 mRNA expression and *SLUG/SNAI2* (**d**), *MMP2* (**e**) and *MMP9* (**f**) mRNA expression. Statistical analysis by Pearson's product moment correlation coefficient. (**g**) Total lysates obtained from PEA1 cells transfected for 72 h with non-targeted control siRNA or TRAP1-directed siRNA were separated by SDS-PAGE and immunoblotted with the indicated antibodies. Numbers indicate densitometric band intensities, calculated by assuming protein levels of the control equal 1. Images are representative of three independent experiments

Cell motility is an important determinant of cancer invasive potential: therefore, we asked whether TRAP1 expression is linked to tumor progression in OC patients. Based on the data of OC tissue biopsies, we analyzed TRAP1 levels in relation to cancer site: results show that low TRAP1 expression is mainly found in peritoneal biopsies localized at distant sites from the primary tumors (Figure 4a). This suggests that TRAP1 low expressing cancers may be more prone to spread and disseminate from the primary site. For this behavior, a key role is played by the activation of EMT gene expression program, which is traditionally considered as a determinant of tumorigenic and metastatic capacity in many cancer types.^28^ Therefore, to confirm a direct role for TRAP1 in regulating the expression of key genes involved in EMT, we analyzed by qPCR: (i) the epithelial marker *E-Cadherin*, (ii) the two major transcription factors controlling *E-Cadherin* expression (*Snail* and *Slug/Snai2*) and (iii) *MMP2* and *MMP9*, two proteins that are also upregulated in EMT and enable invasion and wound healing.^29^ We found that siRNA-mediated silencing of TRAP1 in PEA1 cells leads to upregulation of *Snail1*, *Slug*, *MMP2* and *MMP9* and to downregulation of *E-Cadherin* expression (Figure 4b). *In vivo* relevance of our observations was confirmed by the analysis of TRAP1-related EMT program in our reference database: according to *in vitro* data, we found that lower TRAP1 expression is directly correlated to E-CADHERIN protein levels (Figure 4c) and inversely correlated to *Slug/Snai2* (Figure 4d), *MMP2* (Figure 4e) and *MMP9* (Figure 4f) mRNA levels, thus indicating increased invasive potential. Immunoblot analyses performed in PEA1 cells upon TRAP1 silencing confirmed that TRAP1 and E-CADHERIN protein levels are directly correlated (Figure 4g).

### p70S6K is an EMT inducer in OC

**Figure 5:**
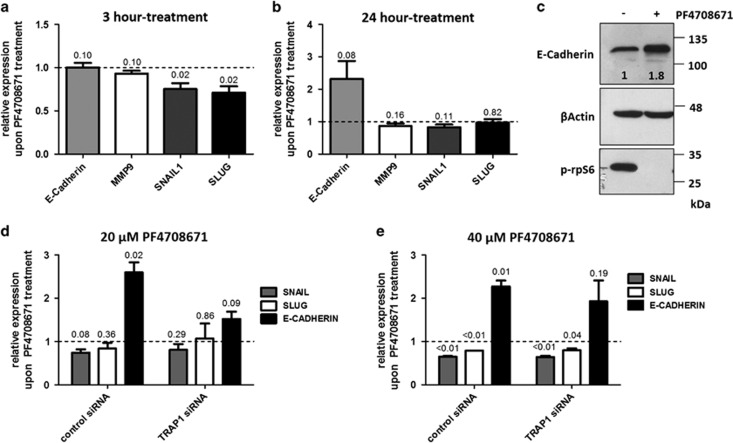
p70S6K regulation of genes involved in EMT. (**a** and **b**) Real-time RT-PCR analysis of *SNAIL, SNAI2/SLUG, MMP9* and *E-Cadherin* expression in PEA1 cells treated with 20 *μ*M PF4708671 for 3 h (**a**) or 24 h (**b**). Data are expressed as mean±S.E.M. from three independent experiments with technical triplicate each. Numbers above bars indicate the statistical significance (*P*-value), based on one-sample *t-*test. Dashed line indicate expression level of the relative control siRNA-transfected cells. (**c**) PEA1 cells were treated with 20 *μ*M PF4708671 for 24 h, total lysates were separated by SDS-PAGE and immunoblotted with the indicated antibodies. Numbers indicate densitometric band intensities, calculated by assuming protein levels of the control equal 1. Images are representative of three independent experiments. (**d** and **e**) Real-time RT-PCR analysis of *SNAIL, SNAI2/SLUG* and *E-Cadherin* expression in PEA1 cells treated with 20 *μ*M (**d**) or 40 *μ*M (**e**) PF4708671 for 3 h (*SNAIL, SLUG* analysis) or 24 h (*E-Cadherin* analysis), following transfection with non-targeted control siRNA or TRAP1-directed siRNA. Data are expressed as mean±S.E.M. from three independent experiments with technical triplicate each. Numbers above bars indicate the statistical significance (*P*-value), based on one-sample *t*-test. Dashed line indicate expression level of the relative untreated cells

**Figure 6:**
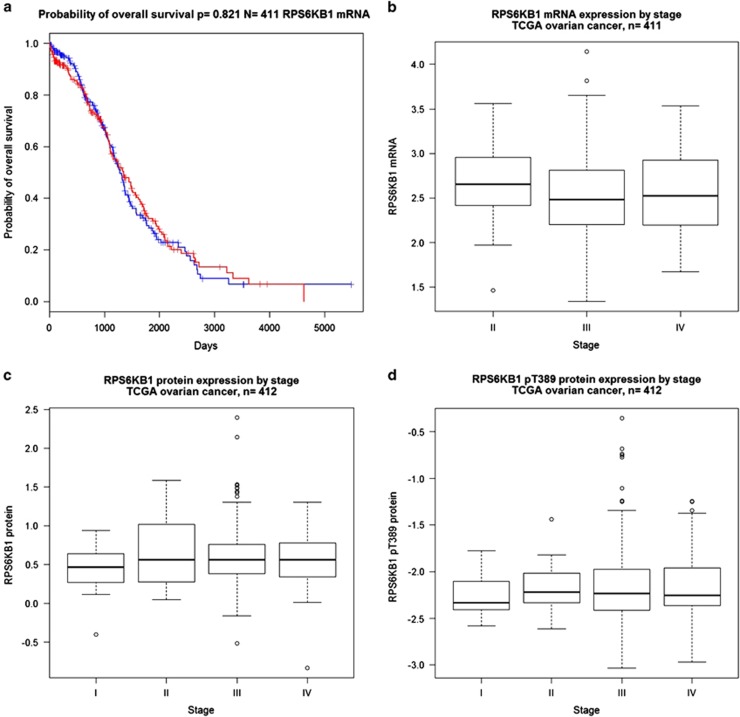
p70S6K expression does not have relevant roles in the advancement of disease. (**a**) Kaplan–Meier estimates of the impact of p70S6K on overall survival (*P*-values determined by the log-rank test). (**b**-**d**) Correlation between p70S6K mRNA (**b**), protein (**c**) or phosphoprotein (**d**) and tumor stage from the TCGA data set (*P*-values determined by the Kruskal–Wallis test)

**Figure 7:**
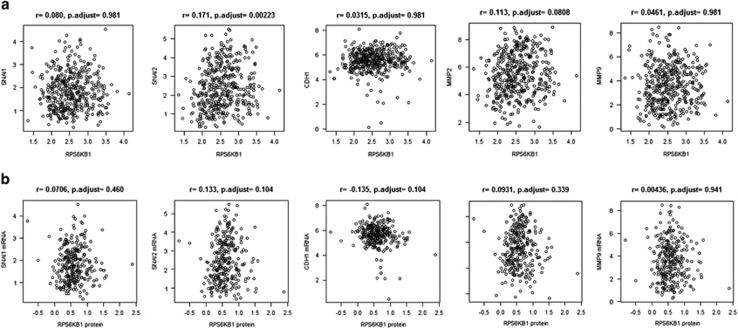
p70S6K does not significantly correlates with EMT genes in OC patients. Correlation between p70S6K mRNA (**a**) or protein (**b**) expression and SNAIL, SLUG/SNAI2, E-Cadherin/CDH1, MMP2 and MMP9 mRNA expression. Statistical analysis by Pearson's product moment correlation coefficient

Our experiments have shown that TRAP1 downmodulation simultaneously leads to p70S6K upregulation and EMT activation; we then questioned whether p70S6K could be directly involved in such regulations, as it has been previously shown that p70S6K induced *Snail* expression in OC cells.^11^ To address this issue, we treated PEA1 cells with the selective p70S6K inhibitor PF4708671 and analyzed the mRNA levels of *E-Cadherin*, *MMP9*, *Snail* and *Slug* by qPCR, 3 and 24 h after treatment. Figure 5 shows that p70S6K inhibition results in early downregulation of the transcription factors *Snail* and *Slug* (Figure 5a), with a consequent increase of *E-Cadherin* mRNA levels at later times (Figure 5b), confirming the role of p70S6K in EMT of these cells. Immunoblots show the significant upregulation of E-CADHERIN protein expression following p70S6K inhibition, which is confirmed by the loss of phosphorylation of the p70S6K target rpS6 (Figure 5c). siRNA-mediated p70S6K silencing yielded similar results (Supplementary Figure 5). Consequently, we performed p70S6K inhibition following siRNA-mediated TRAP1 silencing (Figure 5d). As a result, p70S6K inhibition was less effective in modulating the expression of *Snail, Slug and E-Cadherin* in TRAP1-silenced cells compared with controls; even increasing concentrations of PF4708671 up to 40 *μ*M was not sufficient to revert EMT induced by TRAP1 silencing (Figure 5e). All the PF4708671 concentration used are subtoxic (Supplementary Figure 6). Considering that TRAP1 is upstream p70S6K in their regulation (Figure 1; Supplementary Figure 2), as demonstrated by the absence of TRAP1 regulation upon p70S6K inhibition or silencing (Supplementary Figure 2), these data suggest that EMT regulation in OC is a very complex process in which the contribution of more than one factor and pathway is necessary. The above findings suggest a possible novel clinical implication, that is, OC with reduced TRAP1 expression could be resistant to therapeutic strategies based on the inhibition of the p70S6K pathway. In keeping with this conclusion, we found that, as opposed to TRAP1, p70S6K expression does not have any impact on overall survival (Figure 6a) and there is no correlation between tumor stage and p70S6K mRNA (Figure 6b), protein (Figure 6c) or phosphoprotein (Figure 6d). Accordingly, p70S6K mRNA expression does not correlate with expression of either S*nail, E-Cadherin, MMP2 and MMP9* in a large database of patients (Figure 7a); moreover, the positive correlation between p70S6K and *Slug* mRNA expression is lost when p70S6K protein levels are analyzed (Figure 7b). These observations question the relevance of p70S6K expression/activity in the pathophysiology of OC progression and malignancy, whereas strengthening the importance of TRAP1 as a prognostic marker in this system.

## Discussion

Despite extensive effort, at present survival rates of patients with OC remain poor and OC continues to be a poorly understood disease.^30^ Recent reports have underlined some peculiarities of OC cells at a molecular level, especially linked to metabolic regulations.^31, 32^ Intriguingly, the molecular chaperone TRAP1 has a dominant role in this system: TRAP1 has long been considered uniformly oncogenic in a variety of cancer types,^17^ whereas it inversely correlates with OC stage and grade ^22^ and has a positive impact on patients' survival and response to therapy.^21^ A substantial amount of evidence suggests that the molecular functions exerted by TRAP1 are conserved in a variety of cell models and are not limited to a specific cellular context, for example, regulating the expression of the oncogene BRAF ^33^ and the calcium-binding protein Sorcin ^34, 35^ in both colorectal and breast cancers. Conversely, the effect of such regulations on tumor development and progression are dependent on tumor context. In fact, TRAP1 silencing increases oxidative phosphorylation in several cell lines,^22, 27, 36^ leading to impaired oncogenic potential in osteosarcoma cells,^36^ but to more aggressive phenotype and chemoresistance in OC (Matassa *et al.*;^22^ this study). As such, some data show that higher TRAP1 expression reduces cell motility,^16, 37^ but also increases metastasis in prostate ^26^ and colorectal ^16^ cancers, and confers resistance to conditions normally impairing cell migration.^15, 16^ Accordingly, in this study we show that an inverse correlation between TRAP1 and p70S6K previously found in colorectal cancer is conserved in OC cells and patients, and that this correlation affects cell migration, resulting in increased motility of cells with low TRAP1/high p70S6K levels. Pharmacological suppression of p70S6K significantly decreases cell migration, abolishing the increased motility of TRAP1 knock-down cells.

Cell migration is considered a prerequisite of tumor invasion, but the latter is a complex phenomenon, in which multiple environmental conditions and regulatory mechanisms, such as EMT, are involved. EMT is defined as a functional transition of polarized epithelial cells into mesenchymal cells able to migrate and to secrete components of the extracellular matrix. Carcinoma cells undergoing this transition may induce disease progression by invading and metastasizing.^28^ For this reason, we investigated EMT *in vitro*, upon TRAP1 silencing, and *ex vivo*, by correlating expression of TRAP1 and EMT markers in high-grade serous OC patients. Consistently, low TRAP1 correlated with changes in the expression of genes commonly associated to EMT; accordingly, TRAP1 levels were higher in primary tumor sites than in the peritoneum deposits of advanced diseases and accumulating evidence show that p70S6K could have multiple roles in invasion and metastasis in OC. Thus, depletion of p70S6K in OC cells decreases cell migration,^38^ with significant inhibition of actin cytoskeleton reorganization,^14^ but p70S6K is also involved in EMT activation, via repression of *E-cadherin* through the upregulation of *Snail*;^11^ p70S6K has also recently been proposed as a regulator for the seeding and successful colonization of OC spheroids on the peritoneum.^39^ Accordingly, in our cell models, selective inhibition of p70S6K was able to regulate the expression of EMT genes, showing a function reciprocal to that exerted by TRAP1. Besides, inhibition of p70S6K in TRAP1-silenced cells, expressing higher levels of p70S6K, is not effective in reversing EMT. One of the most relevant result in this study is the absence of correlation of p70S6K and TRAP1 in EMT: differently from the regulation of cell migratory capacity by these two proteins, the obtained results suggest that TRAP1 downmodulation triggers EMT, at least in part, through mechanisms, which are independent from the p70S6K pathway. As a result, in this specific database, p70S6K overexpression seems to be dispensable for the progression of OC, finally displaying no impact on patient survival, advancement of disease and, at a molecular level, expression of genes involved in invasion and EMT.

Notably, albeit EMT is commonly believed to contribute to metastasis, recent studies report that it is conversely dispensable for metastasis and rather contribute to resistance to chemotherapy.^40, 41^ Our recent results showing that downregulation of TRAP1 induces drug resistance in OC cells through metabolic remodeling are in keeping with these data.^22^ More interestingly, our findings suggest a possible novel clinical implication/approach, that is, OC with reduced TRAP1 expression could be resistant to therapeutic strategies based on the inhibition of the p70S6K pathway. However, the role of EMT in drug resistance of OC, although very interesting and novel, will require further in depth studies.

Taken together, these observations support the hypothesis that the metabolic phenotype of the tumor and the nutrient availability in the surrounding microenvironment strongly influence migratory and invasive properties in OC through a modulation of TRAP1 and the expression/activity of p70S6K, thus providing a step forward in the puzzling TRAP1-driven pathophysiology in OC progression and malignancy.

## Materials and Methods

### CNV profiling

The CNV profile was collected by TCGA project for high-grade serous OC patients. The gene-level CNV was estimated using the GISTIC2.0 method ('GISTIC2.0 facilitates sensitive and confident localization of the targets of focal somatic copy number alteration in human cancers') and further thresholded into homozygous deletion (deep deletion), single-copy deletion (shallow deletion), diploid normal copy, low-level copy number amplification or high-level copy number amplification.

### Cell cultures and treatments

The high-grade serous OC cell lines PEA1, PEO1 and PEO14 have been described elsewhere.^42^ Cell line verification was performed by Identifiler kit (Applied Biosystems-Thermo Fisher Scientific, Waltham, MA, USA). All lines were maintained in RPMI 1640 media with 10% fetal bovine serum, penicillin, streptomycin, glutamine at 37 °C/5% CO_2_. Cell lines are routinely monitored in our laboratory by microscopic morphology check.

For wound-healing and gene expression experiments, cells were treated with the selective p70S6K inhibitor PF4708671 (Santa Cruz Biotechnology, Dallas, TX, USA, sc-361288), dissolved in DMSO.

### Transfection procedures

TRAP1 transient silencing was performed with two different siRNAs purchased from Qiagen (Milano, Italy): sense strand: 5′-CGGUCCCUGUACUCAGAAATT-3′ antisense strand: 5′-UUUCUGAGUACAGGGACCGGG-3′ (cat. no. SI00115150); sense strand: 5′-GCUACACCCUGCACUAUAATT-3′ antisense strand: 5′-UUAUAGUGCAGGGUGUAGCGG-3′ (cat. no. SI00115164). p70S6K transient silencing was performed with siRNAs purchased from Qiagen: sense strand: 5′-GAGUUGGACCAUAUGAACUTT-3′ antisense strand: 5′-AGUUCAUAUGGUCCAACUCCC-3′ (cat. no. SI00301721). For control experiments, cells were transfected with a similar amount of nontargeting control siRNA (Qiagen; cat. no. SI03650318). Transient transfections of siRNAs were performed using HiPerFect Transfection Reagent (Qiagen) according to the manufacturer's protocol.

### Western blot analysis

Equal amounts of protein from cell lysates were subjected to SDS-PAGE and transferred to a PVDF membrane (Merck-Millipore, Darmstadt, Germany). Where indicated, protein levels were quantified by densitometric analysis using the software ImageJ.^43^ The following antibodies were used: anti-TRAP1 (sc-13557), anti-*β*-actin (sc-69879) and anti-p70S6K (sc-230), from Santa Cruz Biotechnology; anti-phospho p70 S6 kinase (Thr389) (#9205), anti-E-Cadherin (#3195) and anti-phospho-S6 ribosomal protein (#2215) from Cell Signaling Technology (Danvers, MA, USA).

### RNA extraction and real-time RT-PCR

Total RNA extraction procedures were performed by using TRI Reagent (Sigma-Aldrich, Milano, Italy, product code T9424), following the manufacturer's instruction. The following primers were used for PCR analysis. *TRAP1*: forward 5′-GACGCACCGCTCAACAT-3′, reverse 5′-CACATCAAACATGGACGGTTT-3′ *p70S6K*: forward 5′-ACTTCTGGCTCGAAAGGTGG-3′, reverse 5′-TTGAGTCATCTGGGCTGTCG-3′ *ACTIN*: forward 5′-CCTCACCCTGAAGTACCCA-3′, reverse 5′-TCGTCCCAGTTGGTGACGAT-3′ *E-Cadherin*: forward 5′-CAGCCTGTCGAAGCAGGATT-3′, reverse 5′-TCATCCTCTGGGGGCAGTAA-3′*MMP9*: forward 5′-TTCTACTGGCGCGTGAGTTC-3′, reverse 5′-GCACTGCAGGATGTCATAGGT-3′ MMP2: forward 5′-GCTACGATGGAGGCGCTAAT-3′, reverse 5′-GGGCAGCCATAGAAGGTGTT-3′ *Snail1*: forward 5′-CGAGTGGTTCTTCTGCGCTA-3′, reverse 5′-GGGCTGCTGGAAGGTAAACT-3′ *Slug*: forward 5′-ACAGCGAACTGGACACACAT-3′, reverse 5′-GAGAGGCCATTGGGTAGCTG-3′ *Snail3*: forward 5′-TGACTTATAGTGAGCACCGCC-3′, reverse 5′-ACATAGACGTGTGACATGGGG-3′. When possible, primers were designed to be intron spanning. The reaction conditions were 95 °C for 5 min followed by 45 cycles of 15 s at 95 °C and 1 min at 60 °C. Actin was chosen as the internal control. In PCR analyses performed upon TRAP1 silencing, RNAs were collected 72 h after siRNA transfection.

### Pulse chase

PEA1 cells were incubated in cysteine/methionine-free medium (Sigma-Aldrich) for 30 min followed by incubation in cysteine/methionine-free medium containing 50 *μ*Ci/ml ^35^S-labeled cysteine/methionine (GE Healthcare, Buckinghamshire, UK) for 1 h. After labeling, cells were washed once with culture medium containing excess of unlabeled methionine and cysteine (5 mM each) and incubated further in the same medium. Cells were collected at the indicated time points and lysates were immunoprecipitated with p70S6K antibodies (sc-230) and separated by 10% SDS-PAGE. Proteins were transferred onto a PVDF membrane and analyzed by autoradiography. The same filters were then probed by western blot.

### Wound-healing assay

In order to study the dynamics of wound closure, cells were seeded in monolayer by plating in 12-well plates 2 × 10^5^ cells per well in complete medium; 24 h after plating the cell layer was scratched with sterile pipette tip. Wound healing was followed for 6 h by acquiring digital frames at 2-h intervals with an objective 10x (scale 0.767 pixel/*μ*). Quantitative analysis of wound invasion by cell populations located at the border was performed by measuring the gap area, which was defined by using the wand tool in ImageJ ^43^ and manually refining the selection in the presence of gross errors. The rate of advancement, evaluated as the difference between empty spaces at each time points, was reported as a function of time. Images have been acquired by using the Leica AF6000 Modular System equipped with a 10 × /0.22 objective, and captured by using the Leica LAS AF Lite Software (Leica Microsystems, Milano, Italy). Where indicated, cells were pre-treated with PF4708671 or placed in a glutamine-deprived medium 1 h before performing the scratch.

### Transwell migration assay

Migration was assayed using a modified Boyden chamber (Corning Costar, Corning, NY, USA) containing a polycarbonate membrane filter (6.5 mm diameter, 8 mm pore size) coated with poly-l-lysine. The upper chamber contained cells in RPMI plus 1% FBS in the absence or presence of PF4708671 (20 *μ*M) and the lower chamber contained RPMI plus 10% FBS as a chemoattractant. Cells were incubated for 18 h at 37 °C in 5% CO_2_. Non-migrated cells were scraped off the upper surface of the membrane with a cotton swab. Migrated cells remaining at the bottom surface were fixed with ethanol for 10 min 37 °C and stained with crystal violet (0.5% in 25% methanol) for 10 min at room temperature. Excess staining was washed off by rinsing in water. Staining was eluted with 200 *μ*l of 1% SDS and quantitated by spectrophotometric reading at 570 nm.

### Statistics

Serous OC cases from Tothill data set (Affymetrix U133 plus2.0 microarray, Santa Clara, CA, USA, GSE9891) and TCGA data set (level 3 data, total RNA sequencing and reverse phase protein array) were used to correlate TRAP1 mRNA expression with the expression of other genes. Wilcoxon rank sum test was used to compare gene expression between two groups. Pearson's product moment correlation coefficient was used to correlate the mRNA expression of two genes.

The paired Student's *t*-test was used to establish the statistical significance of differences between gene expression levels in qPCR.

### Patients and study approval

Patients' samples were collected under the Imperial College London Tissue Bank project number R15024 (TRAP1 regulates bioenergetics features, cisplatin resistance and EMT in OC) in accordance with the Imperial College London guidelines. Express written informed consent to use biological specimens for investigational procedures was obtained from all patients. All experimental protocols were approved by Hammersmith and Queen Charlotte's and Chelsea Research Ethics Committee.
