# Supplementary material for: TRAP1 downregulation in human ovarian cancer enhances invasion and epithelial–mesenchymal transition
Source: Cell Death Dis. 2016 Dec 15;7(12):e2522–. doi: 10.1038/cddis.2016.400 (PMC5260997; doi:10.1038/cddis.2016.400)
Supplement: Supplementary Information [file cddis2016400x1.pdf]

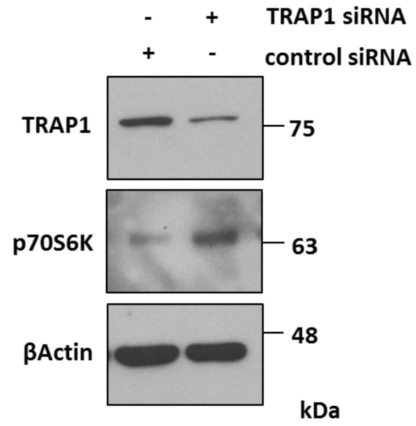

Supplementary Figure 1: TRAP1 level is correlated with lower p70S6K. PEA1 cells were transfected with nontargeting control siRNA or TRAP1-directed siRNA (SI00115164). 72 hours later, total lysates were separated by SDS-PAGE and immunoblotted with the indicated antibodies. Images are representative of three independent experiments.

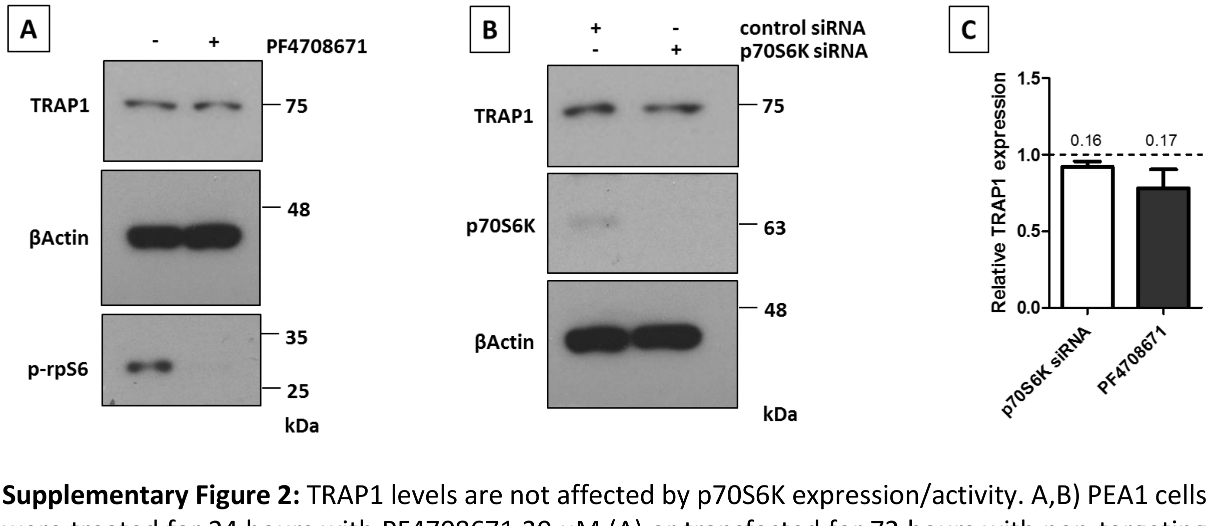

Supplementary Figure 2: TRAP1 levels are not affected by p70S6K expression/activity. A,B) PEA1 cells were treated for 24 hours with PF4708671 20 μM (A) or transfected for 72 hours with non-targeting control siRNA or TRAP1-directed siRNA (B). Subsequently, total lysates were separated by SDS-PAGE and immunoblotted with the indicated antibodies. Images are representative of 3 independent experiments. C) Real-time RT-PCR analysis of TRAP1 mRNAs expression in PEA1 cells upon silencing of p70S6K by siRNA transfection or inhibition by PF4708671 treatment. Data are expressed as mean ± SEM from 3 independent experiments with technical triplicates each. Numbers above bars indicate the statistical significance (p-value), based on the two-tailed Student's t test. Dashed line indicate expression level of the relative control cells.

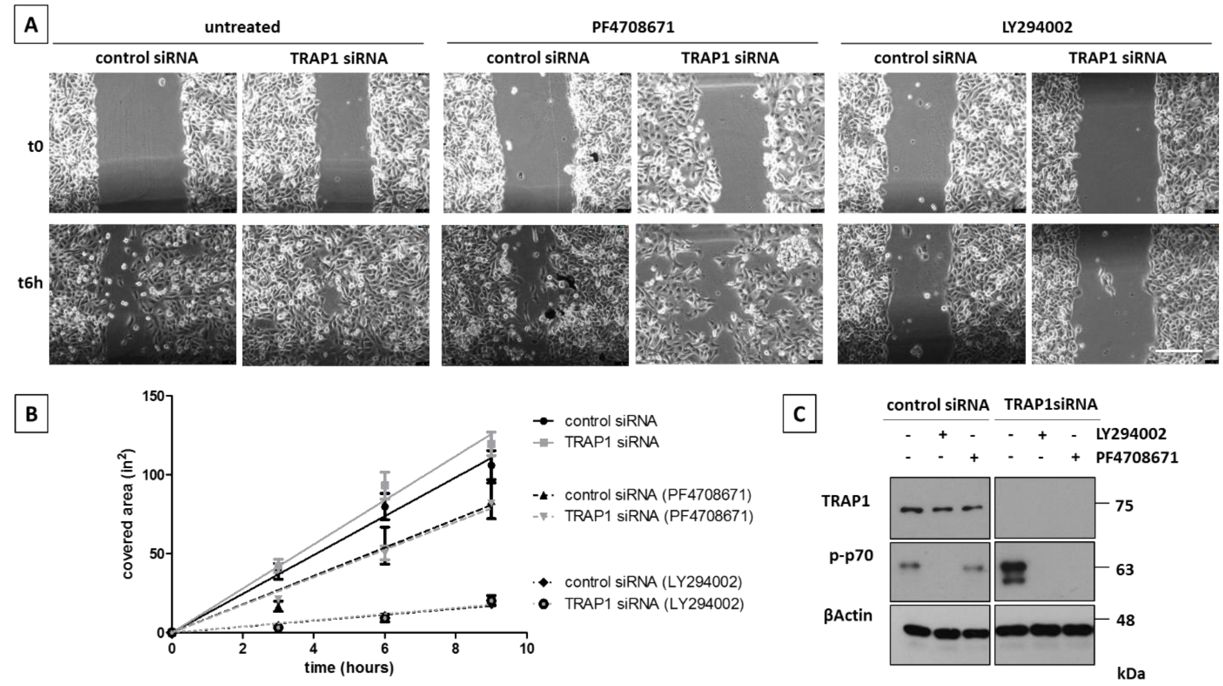

Supplementary Figure 3: TRAP1 affects cell migration through p70S6K. A,B) Time-lapse acquisition of untreated and PF4708671/ LY294002 treated PEA1 cells transfected for 72h with non-targeted control siRNA or TRAP1-directed siRNA, immediately after the wound (t0) and after 6 hours (t6h). Scale bar, 500 µm. The rate of advancement, evaluated as the difference between empty space at each time point, is reported as a function of time in panel B for cells transfected with control siRNA (black) and TRAP1-directed siRNA (grey). Dashed lines are used for PF4708671 treated cultures, dotted line for LY294002 treated cultures and solid lines for untreated cultures. Data are expressed as mean ± SEM from four independent experiments. Lines have been fitted with linear regression. C) PEA1 cells transfected for 72h with non-targeted control siRNA or TRAP1-directed siRNA, then treated with 20 µM PF4708671 or 10 µM LY294002 for 1 hour. Total lysates were separated by SDS-PAGE and immunoblotted with the indicated antibodies. Images are representative of three independent experiments.

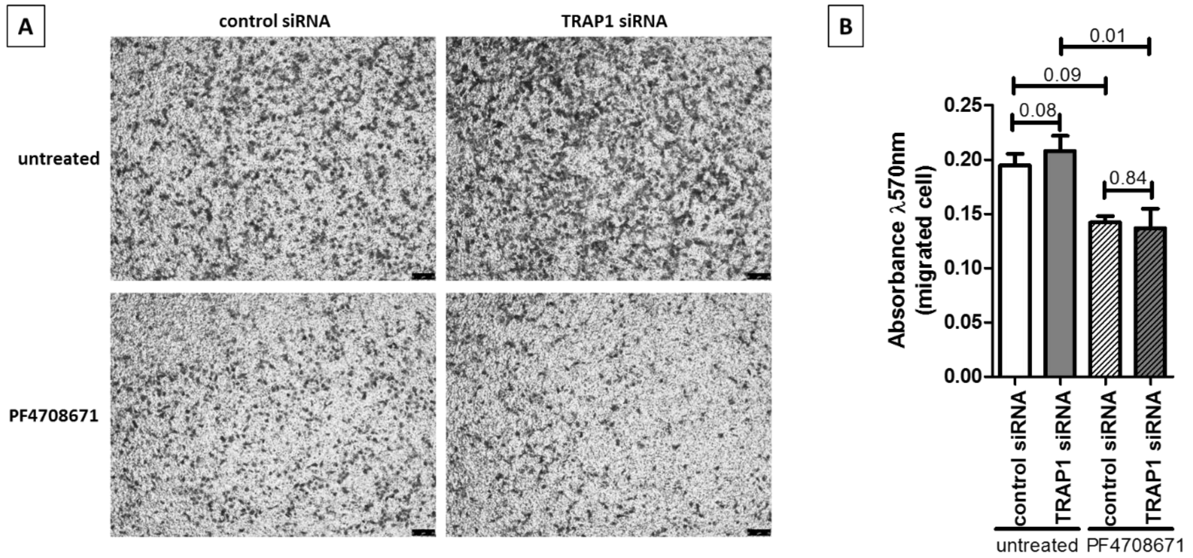

Supplementary Figure 4: TRAP1 affects cell migration through p70S6K. Representative images (A) of transwell migration assay using 10% FBS as chemoattractant. Scale bar: 75 µm. The data summary (B) shows absorbance of the Crystal violet staining eluted by migrated cells and quantitated by spectrophotometric reading at 570 nm. Data are expressed as mean ± SEM from three independent experiments. Numbers above bars indicate the statistical significance (p-value), based on Student's t test.

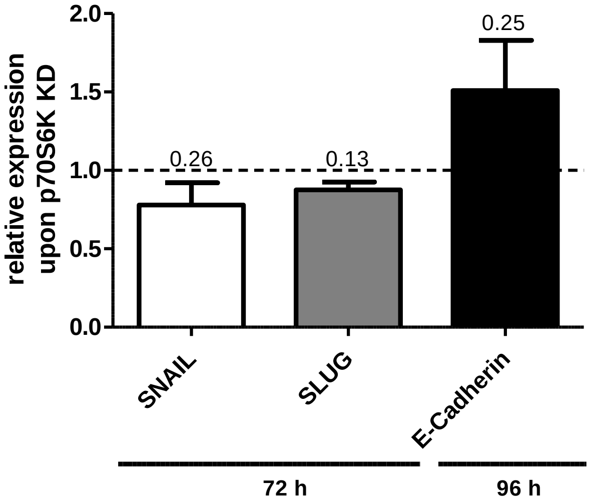

Supplementary Figure 5: p70S6K silencing increases E-Cadherin expression. Real-time RT-PCR analysis of Snail, Slug and E-Cadherin expression in PEA1 cells 72h and 96h after transfection with non-targeted control siRNA or p70S6K-directed siRNA. Data are expressed as mean ± SEM from 3 independent experiments with technical triplicates each. Numbers above bars indicate the statistical significance (p-value), based on one-sample t test. Dashed line indicate expression level of the relative control siRNA-transfected cells.

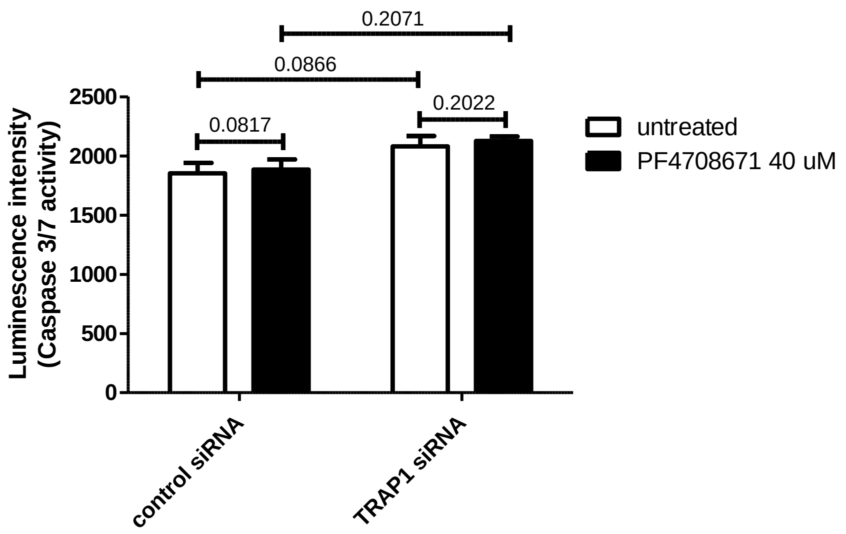

Supplementary Figure 6: PEA1 cells were treated with 40 µM PF4708671 for 24 h, then apoptosis was measured by caspase 3/7 activity luminescent assay. Data are expressed as mean ± S.E.M. from three independent experiments with technical triplicates each. Numbers above bars indicate the statistical significance (P-value), based on the two-tailed Student's t-test.
